# Charlson syndrome index predicted survival in pancreatic cancer patients received immunotherapy

**DOI:** 10.3389/fimmu.2025.1487318

**Published:** 2025-02-03

**Authors:** Nan Zhang, Shiyun Chen, Yue Shi, Zhikuan Wang, Ru Jia, Guanghai Dai

**Affiliations:** ^1^ Medical School of Chinese PLA, Beijing, China; ^2^ Department of Medical Oncology, the First Medical Center, Chinese PLA General Hospital, Beijing, China; ^3^ Department of Medical Oncology, the Fifth Medical Center, Chinese PLA General Hospital, Beijing, China

**Keywords:** pancreatic cancer, immune checkpoint inhibitors, Charlson syndrome index, survival, immunotherapy combined therapy

## Abstract

**Objective:**

The Charlson Comorbidity Index (CCI) is a widely utilized assessment tool for evaluating the mortality rate among patients with chronic diseases and tumors. Currently, there is a dearth of research investigating the correlation between CCI and survival rates in advanced pancreatic cancer patients received immunotherapy. Therefore, this study aims to elucidate the association between CCI and survival rates in real-world settings for pancreatic cancer patients received immunotherapy.

**Methods:**

A total of 104 patients with advanced pancreatic cancer who received immunotherapy at the General Hospital of the People’s Liberation Army between September 2015 and September 2020 were included in this study. The patients were categorized into two groups based on their Charlson Comorbidity Index (CCI) scores: low CCI group (CCI <7) and high CCI group (CCI ≥7). The statistical analysis focused on examining the correlation between CCI score and survival outcome.

**Results:**

The high CCI group exhibited significantly lower overall survival (OS) and progression-free survival (PFS) compared to the low CCI group (p<0.05). The median OS for the high CCI and low CCI groups were 7.82 and 44.17 months, respectively, while the median PFS were 2.40 and 6.40 months, respectively. Multivariate analysis revealed that high CCI was independently risk factor for both OS (HR=2.801, 95%CI: 1.433-5.472, p=0.003) and PFS (HR=2.546, 95%CI: 1.389-4.668, p=0.003).

**Conclusion:**

The CCI score serves as a significant independent predictive indicator for advanced pancreatic cancer patients received immunotherapy.

## Introduction

Pancreatic cancer (PC) is a highly malignant digestive system tumor with extremely poor prognosis, often diagnosed in advanced stages and progressing rapidly (1, 2). Currently, the treatment of PC still mainly relies on chemotherapy, with a median overall survival of less than 1 year (3–5). Although immunotherapy for PC has been continuously explored, it has not improved the overall prognosis compared to chemotherapy alone (6).

PC patients are often accompanied by other chronic diseases, and a higher number of comorbidities indicates lower treatment efficacy and shorter overall survival. The Charlson Comorbidity Index (CCI) is a widely used indicator that assesses the overall health status of patients by calculating scores and weights for chronic diseases. It has been extensively studied in various tumor types, including prostate cancer (7, 8), colorectal cancer (9), pancreatic cancer (10) etc., but there are no reports on the predictive role of the CCI index in immunotherapy for PC.

Therefore, we conducted an analysis of real-world data to assess the prognostic significance of CCI scores in PC patients received immunotherapy.

## Patients and methods

Patients diagnosed with advanced PC who received treatment with PD-1 inhibitors (including nivolumab, pembrolizumab, sintilimab) at the First Medical Center of the Chinese People’s Liberation Army General Hospital (Beijing, China) from September 2015 to September 2020. The inclusion criteria were as follows: confirmed histopathological diagnosis of locally advanced or metastatic PC; aged 18 years or older; Eastern Cooperative Oncology Group (ECOG) status of 0–1; have undergone two–lines treatment at least including chemotherapy combine with PD-1 inhibitors; have assessable lesions through imaging scans (CT) and blood tests during immunotherapy. The exclusion criteria included: pancreatic neuroendocrine tumors, benign pancreatic conditions, no prior use of PD-1 inhibitors, or lack of imaging examinations.

Clinical data were electronically retrieved from the medical records of the PLA General Hospital Registry. Essential clinical characteristics of patients, including age, gender, tumor differentiation grade, smoking and alcohol history, past medical history, other comorbidities, concurrent medication use, pre-treatment tumor marker CA19-9 levels, as well as metastatic sites and quantities, were gathered. Patient data were initially registered and subsequently categorized based on variable types. Continuous variables included age and CA19-9 levels, whereas categorical variables encompassed gender, smoking history, drinking history, degree of differentiation, and others. Survival status was assessed via telephone follow-up at the end of the observation period. All data were verified for accuracy and reliability.

This study was authorized by the Ethics Committee of the Chinese People’s Liberation Army General Hospital and conducted in accordance with the principles outlined in the Helsinki Declaration.

The Charlson Comorbidity Index (CCI) score was calculated based on a comprehensive review of clinical data considering patients’ past medical history and comorbidity status, drawing on ICD-10 recordings of 17 common chronic medical conditions (11) (see **Appendix 1**). Efficacy evaluation was performed on all patients received who immunotherapy according to RECIST 1.1 criteria, followed by monitoring their survival status including overall survival (OS) and progression-free survival (PFS).

## Statistical analysis

IBM SPSS version 26.0 and R studio software were used to perform statistical analysis. The Kaplan-Meier method was utilized to analyze OS and PFS, and the differences were evaluated by the log-rank test. The clinical characteristics of patients were compared using Chi-squares or Fisher’s exact test. The optimal CCI score cut-off values for OS and PFS were determined by ROC analysis. The Cox proportional hazards regression model was used for univariate and multivariate analysis. Multivariate analysis was performed on covariates that showed a significant correlation with OS and PFS in univariate analyses. R Studio software was used for generating figures. All statistical tests were two-sided, and p<0.05 was considered statistically significant.

## Results

### Clinical characteristics of patients

A total of 104 patients with advanced PC who had received PD-1 inhibitor therapy were included in this study. The clinical indicators of the patients were recorded, and the Charlson Comorbidity Index (CCI) was calculated (see **Table 1**). The majority of patients (76.9%) received a combination treatment of PD-1 inhibitors and chemotherapy as their first-line therapy. In this group, the median age was 56 years, with 73 cases (70.2%) being male, 59 cases (56.7%) having poorly differentiated tumors, and 90 cases (86.5%) having ≤2 sites of metastasis; before treatment, CA19-9 levels were elevated in 77 individuals (74.0%).

**Table 1:** Characteristics of patients with advanced PC.

Characteristics	No. of Patients (%)
Age, years	
<60	69 (66.3%)
≥60	35 (33.7%)
Sex	
Female	31 (29.8%)
Male	73 (70.2%)
Smoking history	
Never smoke	67 (64.4%)
Smoke	37 (35.6%)
Histological differentiating degree	
Moderate and high differentiation	45 (43.3%)
Low differentiation	59 (56.7%)
Metastatic sites	
0-2	90 (86.5%)
>2	14 (13.5%)
Baseline CA-199	
>Normal level	77 (74.0%)
≤Normal level	23 (22.1%)
Missing	4 (3.8%)
Combined with other drugs	
Yes	95 (91.3%)
No	9 (8.7%)
Line of immunotherapy	
<2	80 (76.9%)
≥2	24 (23.1%)
Charlson comorbidity index	
<7	46 (44.2%)
≥7	58 (55.8%)

The median Charlson Comorbidity Index (CCI) for patients was 7 (range 3-14) in this study. Patients were divided into the high CCI group (CCI≥7) and the low CCI group (CCI<7). The clinical factors between the two groups were approximately equilibrated at baseline, exhibiting no statistically significant disparities (**Table 2**).

**Table 2:** Differences of patients’ characteristics between the CCI ≥7 group and CCI<7 group.

Characteristics	No. of Patients (%)		P value
	CCI ≥7	CCI <7	
	(n = 62)	(n =42)	
Age, years			
<60	37 (63.8%)	32 (69.6%)	
≥60	21 (36.2%)	14 (30.4%)	
Sex			0.901
Female	17 (29.3%)	14 (30.4%)	
Male	41 (70.7%)	32 (69.6%)	
Smoking history			0.072
Never smoke	33 (56.9%)	34 (73.9%)	
Smoke	25 (43.1%)	12 (26.1%)	
Histological differentiating degree			0.217
Moderate and high differentiation	22 (37.9%)	23 (50%)	
Low differentiation	36 (62.1%)	23 (50%)	
Metastatic sites			0.065
0-2	47 (81%)	43 (93.5%)	
>2	11 (19%)	3 (6.5%)	
Baseline CA-199			0.136
≤Normal level	10 (17.5%)	13 (30.2%)	
>Normal level	47 (82.5%)	30 (69.8%)	
Missing	1	3	
Combined with other drugs			0.736
Yes	52 (89.7%)	43 (93.5%)	
No	6 (10.3%)	3 (6.5%)	
Line of immunotherapy			0.220
<2	42 (72.4%)	38 (82.6%)	
≥2	16 (27.6%)	8 (17.4%)	

### ROC analysis predicted cut-off values of Charlson comorbidity index score for OS and PFS

The ROC curve determined the cut-off value of the Charlson score for OS > 12m.

**Figure 1:**
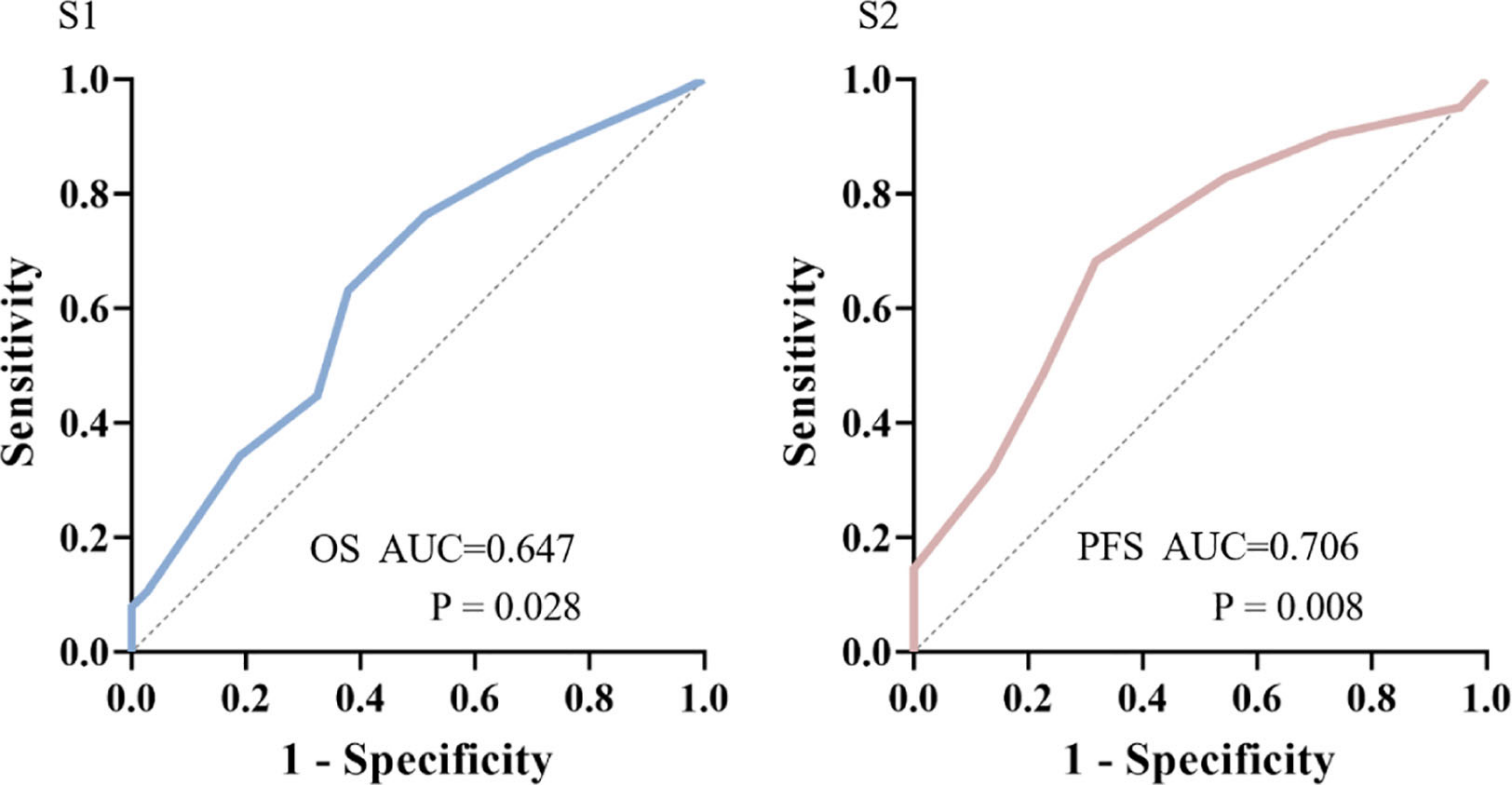
ROC analysis predicted cut-off values of Charlson Comorbidity Index (CCI) score for OS>12m(S1) or PFS >6m(S2).

At the cut-off value of 6.5, the maximum Youden index is 0.365, with both sensitivity and specificity at 68.3%. The AUC is 0.647 (95% CI: 0.522-0.772, p=0.028) (**Figure 1** S1).

The ROC curve determined the cut-off value of the Charlson score for PFS > 6m.

At the cut-off value of 6.5, the maximum Youden index is 0.253, with sensitivity at 63.2% and specificity at 62.2%. The AUC is 0.706 (95% CI: 0.572-0.840, p=0.008) (**Figure 1** S2).

Since the CCI scores were all integers and the median of CCI was 7, we divided the patients into the high CCI group (CCI≥7) and the low CCI group (CCI<7) for survival analysis.

### Association between CCI and prognosis

#### Effect of the CCI indicator on OS

**Figure 2:**
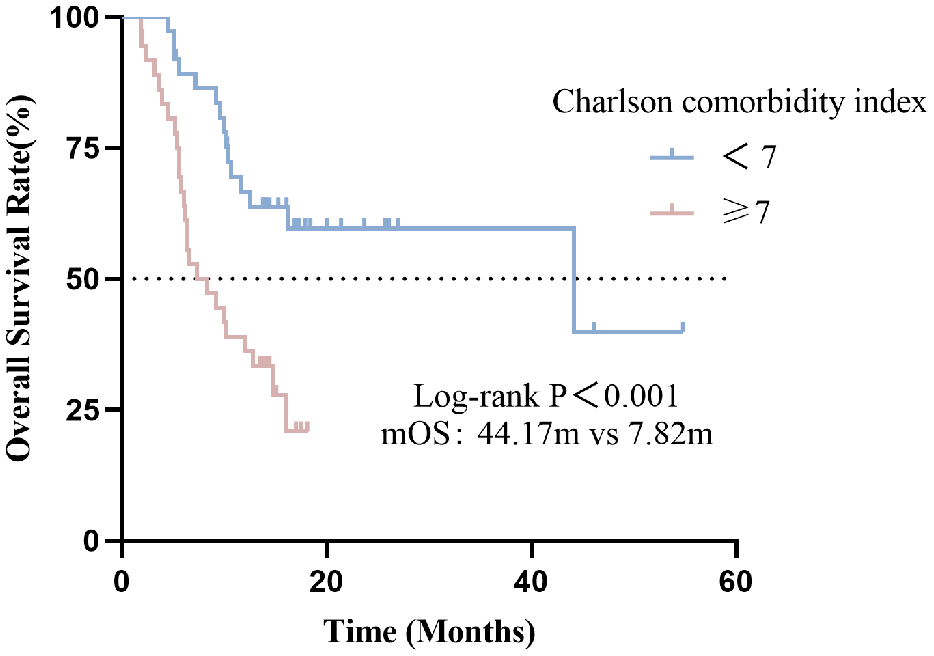
Association between CCI indicator with OS.

In this cohort, the median OS was 12.10 months (95% CI: 7.40-16.80). Compared with the high CCI group, the low CCI group (CCI<7) had significantly better overall survival than the high CCI group (CCI≥7) (p <0.001) (**Figure 2**). The median OS for patients with CCI<7 and CCI≥7 were 44.17 and 7.82 months, respectively (p<0.001).

**Figure 3:**
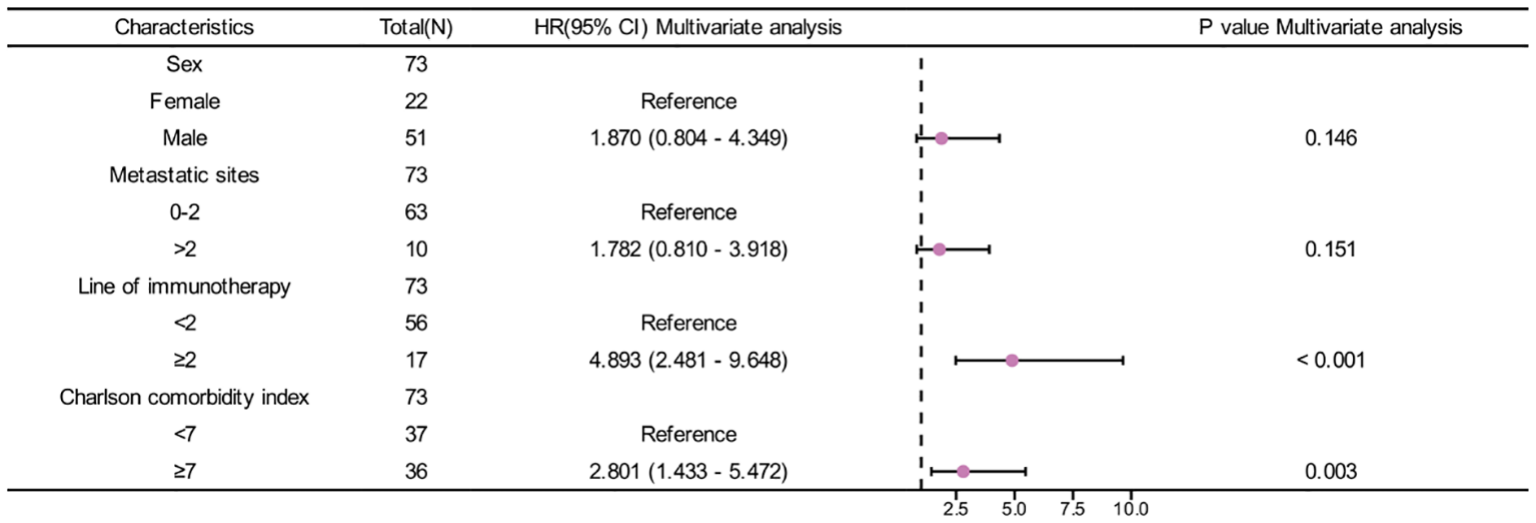
Forest plot of CCI index and OS.

In univariate analysis, sex, metastatic sites, line of immunotherapy ≥2 and CCI index were correlated with OS (p <0.05) (**Table 3**). In multivariate analysis, CCI ≥7 was an independent risk factor for poor prognosis (HR=2.801, 95%CI:1.433-5.472, p=0.003). Additionally, Line of immunotherapy ≥2 was also an independent risk factor for poor prognosis (**Table 3**, **Figure 3**).

**Table 3:** Univariate and multivariate analysis for OS in patients treated with ICIs.

Characteristics	HR(95% CI) Univariate analysis	P value Univariate analysis	HR(95% CI) Multivariate analysis	P value Multivariate analysis
Age		0.745		
<60	Reference			
≥60	1.110 (0.591 - 2.086)			
Sex		0.021		0.146
Female	Reference		Reference	
Male	2.637 (1.161 - 5.991)		1.870 (0.804 - 4.349)	
Smoking history		0.329		
Never smoke	Reference			
Smoke	1.376 (0.725 - 2.611)			
Histological differentiating degree		0.328		
Moderate and high differentiation	Reference			
Low differentiation	1.376 (0.726 - 2.607)			
Metastatic sites		0.020		0.151
0-2	Reference		Reference	
>2	2.534 (1.158 - 5.545)		1.782 (0.810 - 3.918)	
Baseline CA-199		0.358		
≤Normal level	Reference			
>Normal level	1.470 (0.647 - 3.341)			
Combined with other drugs		0.984		
Yes	Reference			
No	1.013 (0.287 - 3.581)			
Line of immunotherapy		< 0.001		< 0.001
<2	Reference		Reference	
≥2	5.401 (2.787 - 10.469)		4.893 (2.481 - 9.648)	
Charlson comorbidity index		0.001		0.003
<7	Reference		Reference	
≥7	2.940 (1.521 - 5.683)		2.801 (1.433 - 5.472)	

OS, overall survival; HR, hazard ratio.

#### Effect of the CCI indicator on PFS

**Figure 4:**
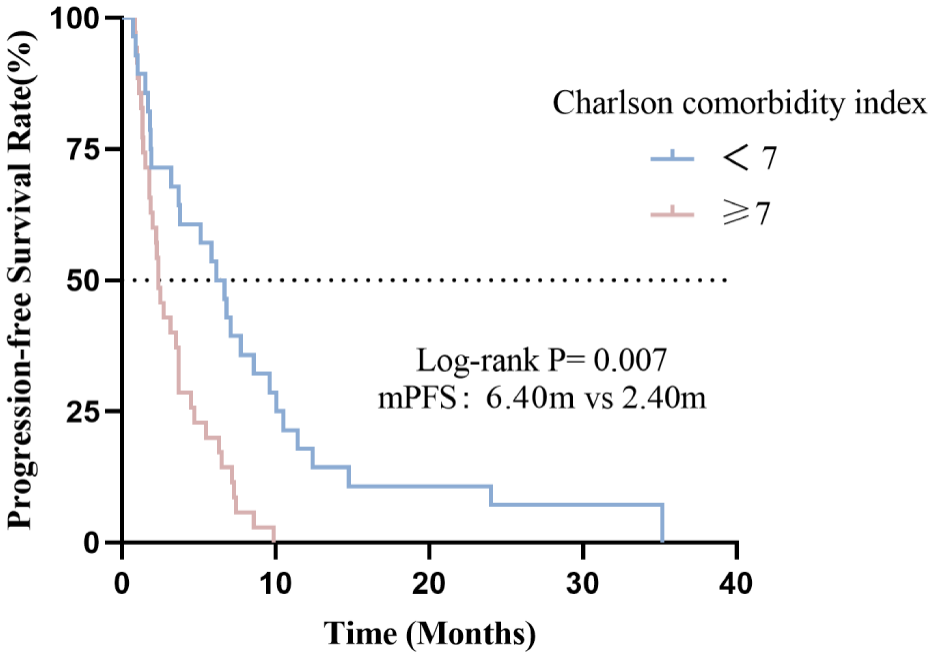
Association between CCI indicator with PFS.

In this cohort, the median PFS was 3.50months (95% CI: 2.40-4.60). Compared with the high CCI group, the low CCI group (CCI <7) had significantly better progression-free survival than the high CCI group (CCI≥7) (p=0.007) (**Figure 4**). The median PFS for patients with CCI<7 and CCI≥7 were 6.40 and 2.40 months, respectively (p=0.007).

**Figure 5:**
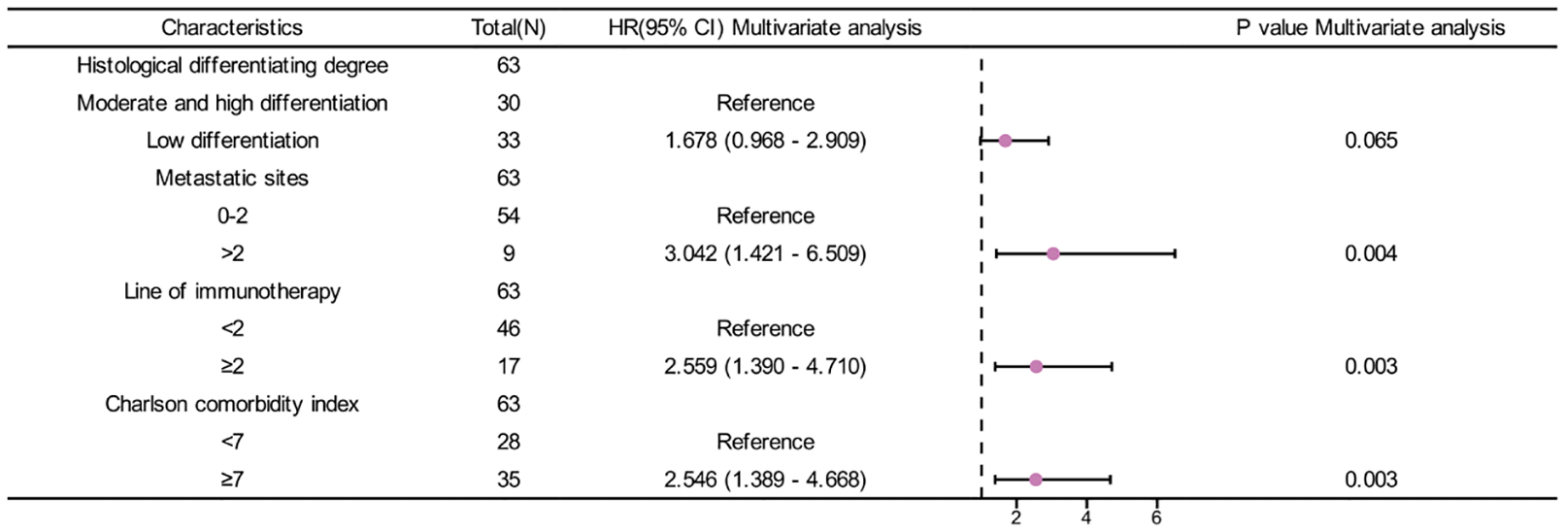
Forest plot of CCI index and PFS.

In univariate analysis, Histological differentiation degree(p=0.012), metastatic sites(p<0.001), line of immunotherapy≥2 (p=0.003) and CCI index (p<0.001) were correlated with OS (**Table 4**). In multivariate analysis, CCI≥7 (HR=2.546, 95%CI: 1.389-4.668, p=0.003) was an independent risk factor for poor prognosis. Additionally, metastatic sites>2(HR=3.042, 95%CI: 1.421-6.509, p=0.004) and line of immunotherapy≥2 (HR=2.559, 95%CI: 1.390-4.710, p=0.003) were also an independent risk factor for poor prognosis (**Table 4**, **Figure 5**).

**Table 4:** Univariate and multivariate analysis for PFS in patients treated with ICIs.

Characteristics	HR(95% CI) Univariate analysis	P value Univariate analysis	HR(95% CI) Multivariate analysis	P value Multivariate analysis
Age		0.403		
<60	Reference			
≥60	0.797 (0.468 - 1.356)			
Sex		0.064		
Female	Reference			
Male	1.723 (0.969 - 3.061)			
Smoking history		0.299		
Never smoke	Reference			
Smoke	1.318 (0.782 - 2.221)			
Histological differentiating degree		0.012		0.065
Moderate and high differentiation	Reference		Reference	
Low differentiation	2.026 (1.166 - 3.520)		1.678 (0.968 - 2.909)	
Metastatic sites		< 0.001		0.004
0-2	Reference		Reference	
>2	3.913 (1.827 - 8.382)		3.042 (1.421 - 6.509)	
Baseline CA-199		0.066		
≤Normal level	Reference			
>Normal level	1.839 (0.961 - 3.520)			
Combined with other drugs		0.610		
Yes	Reference			
No	0.797 (0.334 - 1.904)			
Line of immunotherapy		0.003		0.003
<2	Reference		Reference	
≥2	2.413 (1.354 - 4.300)		2.559 (1.390 - 4.710)	
Charlson comorbidity index		< 0.001		0.003
<7	Reference		Reference	
≥7	2.711 (1.525 - 4.819)		2.546 (1.389 - 4.668)	

PFS, progression-free survival; HR, hazard ratio.

### Risk score assess a patient’s risk of death

**Figure 6:**
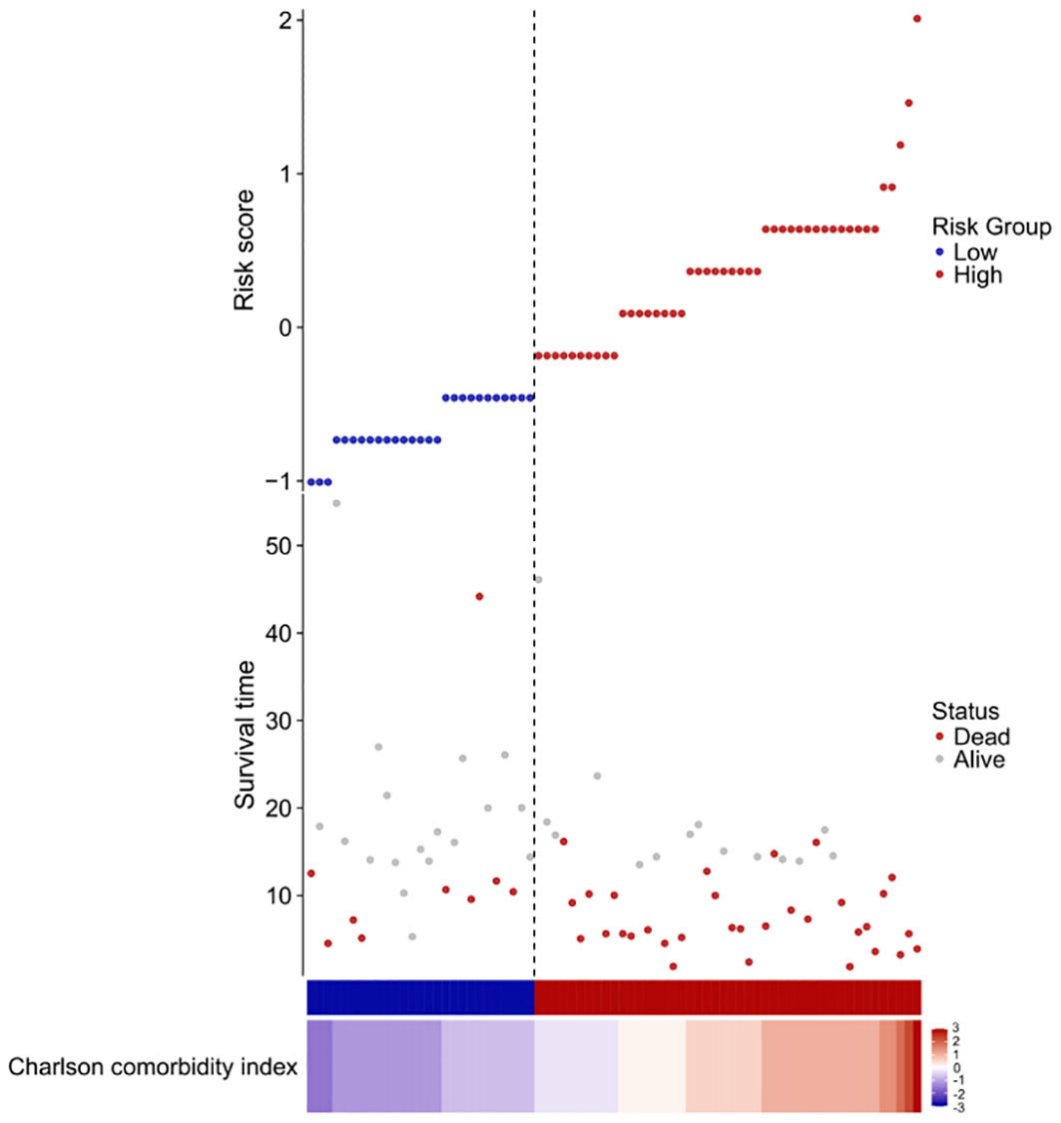
Risk factor diagram of CCI indicator. Charlson comorbidity index and risk score, survival time distribution in our cohort by z-score. Here, red indicates higher score, while light blue indicates lower expression. The risk scores for all patients are plotted in ascending order and marked as low risk (blue) or high risk (red), as divided by the threshold (vertical black line). The risk score threshold is 7. The bars indicate the survival times for each case. Red bars represent dead cases and blue bars represent alive during observation.

We constructed a risk score using the regression coefficients from this model and manually selected a suitable threshold at the 50th percentile. The risk score, survival time, and Charlson comorbidity index for each patient are shown in **Figure 6**.

As can be seen from the figure, the survival time of low-risk individuals is longer than that of high-risk individuals, and the mortality rate among high-risk individuals is significantly higher than that among low-risk individuals.

## Discussion

Immunotherapy is currently considered a promising approach in anti-tumor treatment, as it has demonstrated improved overall prognosis for patients with various types of cancer (12–14). It has also identified specific biomarkers and clinical factors, such as PD-L1 expression levels and MSI status, that can effectively predict the efficacy of immunotherapy. However, unfortunately, PC has not shown significant survival benefits from PD-1 inhibitors (15, 16). Therefore, it is crucial to identify factors associated with immunotherapy in order to select the population that will derive maximum benefit from this treatment modality, which holds immense clinical significance.

Our study aimed to analyze the correlation between Charlson Comorbidity Index (CCI) scores and prognosis in advanced PC patients received immunotherapy in real-world settings. The findings revealed that the CCI ≥7 and line of immunotherapy ≥2 were independent risk factors affecting PFS and OS in late-stage disease. These results are both expected and thought-provoking. The median OS of 44.17 months, derived from the Kaplan-Meier curve, is not the true median OS for all patients and should be used as a partial reference. This may be due to patients in the low CCI group (CCI < 7) remaining alive at follow-up completion and the number of patients receiving immunotherapy. Future studies with larger sample sizes and extended follow-up periods can verify this.

As we know, the CCI score is a widely used comorbidity index initially proposed by Charlson et al. (11), with subsequent studies demonstrating its impact on survival rates and prognosis for specific solid tumors such as HER2-positive breast cancer in elderly females (17), sinonasal squamous cell carcinoma (18), gastric cancer (19), renal cell carcinoma (20)and surgically treated PC (21). In all these cases, higher CCI groups have consistently exhibited worse overall prognosis compared to lower CCI groups.

Unlike other types of cancer, PC patients generally exhibit higher CCI scores. The presence of metastasis alone contributes a weightage of 6 points to the CCI score. Moreover, most PC patients often present with one or more chronic comorbidities, and the number and severity of these comorbidities impact the treatment and prognosis of PC. A greater number of comorbidities typically indicates a reduced likelihood of receiving treatment and an increased non-tumor-related mortality rate. Tomonari Asano et al.’s study (21) also focused on operable PC patients, yielding similar results that established a high cutoff point for CCI at 4 points. The proportion of patients who received chemotherapy in the low CCI group was significantly higher than that in the high CCI group (87% vs. 69%, P < 0.0001). The overall survival rate in the low CCI group was significantly higher than that in the high CCI group (p= 0.047). Multivariate analysis indicated that high CCI was a predictive factor for lower survival rates (P = 0.024). In the high CCI group, patients with a high relative dose intensity (RDI) of adjuvant chemotherapy had significantly better recurrence-free survival and OS compared to those with a low RDI (both P < 0.0001).

Our study reached consistent conclusions while additionally discovering that both stratification based on CCI scores and line of immunotherapy impacted patient survival, including overall survival and progression-free rates. Patients who received immunotherapy (line of immunotherapy ≥2) exhibited poorer overall efficacy and prognosis compared to those who received it earlier. This may be attributed to early recipients having better physical conditions, synergistic anti-tumor effects from immunotherapy, as well as increased opportunities for tailing off effects to manifest-factors enabling patients to derive greater benefits from immunotherapy treatments. This likely explains why early use of immunotherapy is recommended for patients. Furthermore, there exists a correlation between the number of metastatic sites and patient’s PFS, indicating that a higher tumor burden increases the likelihood of rapid progression after undergoing immunotherapy treatments. We also reviewed the correlation between the CCI scores of solid tumor patients who received immunotherapy and the occurrence of immune-related adverse events (irAEs) (22, 23). The results showed a significant association between CCI scores and irAEs in lung cancer and renal cancer patients. However, no significant relationship was found in malignant melanoma patients. Therefore, it is considered that CCI can be used to predict irAEs, but specific comorbidity indices, including autoimmune diseases, should be combined to provide a more reliable indication.

Age is a critical determinant in the calculation of CCI scores, and it is advisable to make adjustments for age (ACCI) when evaluating cancer mortality and survival rates in elderly cancer patients using the CCI tool. The inter-rater reliability of CCI among elderly cancer patients demonstrates robustness (24), with values ranging from 0.74 to 0.945. Furthermore, findings affirm that elevated CCI serves as an independent prognostic risk factor for elderly cancer patients (25). Within this specific patient cohort, where the median age was 56 years, univariate analysis also indicates that age does not present as a significant risk factor for survival outcomes. Hence, when advocating for the utilization of the CCI index in prognostic assessments, consideration should be given to incorporating age factors to enhance predictive accuracy. Partial meta-analysis also indicates that there is no significant difference in overall survival and progression-free survival between the younger and elderly groups receiving anti-PD-1/anti-PD-L1 and CTLA-4 therapy (26, 27). However, PD-L1 expression increases with age, and higher PD-L1 expression is often associated with better immunotherapy outcomes (28).

According to SEER 22 and Cancer statistics, pancreatic cancer predominantly affects individuals aged 60-70, accounting for 40% of cases. However, in this study of 104 patients, only 35 (33.7%) were over 60. This discrepancy may be due to the study’s single-center, small-sample retrospective nature, which limits its representation of all pancreatic cancer patients. Additionally, recent trends show an increase in younger patients (29, 30). Improved diagnostic and treatment capabilities across different regions and hospitals also influence patient data (31). Despite variations in age composition, real-world data indicate no significant relationship between age and immune treatment response. Middle-aged patients (aged < 60) with a CCI < 7 benefit more from immunotherapy, showing prolonged OS and PFS. Future large-scale or prospective studies are needed to confirm these findings.This study has several limitations. Firstly, it is a retrospective single-center study with a limited sample size, and the CCI scores were derived from reviewed cases, potentially introducing selection bias, recall bias, and confounding factors.

The stratification of CCI was based on the median score of patients in this cohort. Given that most patients in this study had advanced pancreatic cancer with metastasis, the conclusions are likely more applicable to individuals with similar conditions. Nonetheless, we have presented a straightforward, convenient, and non-invasive approach utilizing a patient’s chronic disease index and disease burden to aid in identifying late stage PC patients who could benefit from PD-1 inhibitor therapy in clinical settings.

## Conclusions

Our findings confirm that the CCI can predicted the efficacy and prognosis of advanced PC in patients who received PD-1 inhibitors, and also assist physicians in making more optimal clinical treatment decisions. The CCI score is crucial for future immunotherapy in PC.

## Data Availability

The raw data supporting the conclusions of this article will be made available by the authors, without undue reservation.

## References

[1] Siegel RL Giaquinto AN Jemal A. Cancer statistics, 2024. CA: Cancer J For Clin. (2024) 74:12–49. doi: 10.3322/caac.21820 38230766

[2] Hidalgo M. Pancreatic cancer. N Engl J Med. (2010) 362:1605–17. doi: 10.1056/NEJMra0901557 20427809

[3] Conroy T Desseigne F Ychou M Bouché O Guimbaud R Bécouarn Y. FOLFIRINOX versus gemcitabine for metastatic pancreatic cancer. N Engl J Med. (2011) 364:1817–25. doi: 10.1056/NEJMoa1011923 21561347

[4] Von Hoff DD Ervin T Arena FP Chiorean EG Infante J Moore M. Increased survival in pancreatic cancer with nab-paclitaxel plus gemcitabine. N Engl J Med. (2013) 369:1691–703. doi: 10.1056/NEJMoa1304369 PMC4631139 24131140

[5] Wainberg ZA Melisi D Macarulla T Cid RP Chandana SR De La Fouchardière C. NALIRIFOX versus nab-paclitaxel and gemcitabine in treatment-naive patients with metastatic pancreatic ductal adenocarcinoma (NAPOLI 3): a randomised, open-label, phase 3 trial. Lancet. (2023) 402:1272–81. doi: 10.1016/s0140-6736(23)01366-1 PMC11664154 37708904

[6] Wainberg ZA Hochster HS Kim EJ George B Kaylan A Chiorean EG. Open-label, phase I study of nivolumab combined with nab -paclitaxel plus gemcitabine in advanced pancreatic cancer. Clin Cancer Res. (2020) 26:4814–22. doi: 10.1158/1078-0432.Ccr-20-0099 32554514

[7] Cross DS Islam R Mullins CD Hussain A Jacobson C Foth W. The effects of age, stage, treatment, and Charlson comorbidity index (CCI) on quality of life after prostate cancer diagnosis in a single cohort. J Clin Onco. (2013) 31:242–42. doi: 10.1200/jco.2013.31.6_suppl.242

[8] Jankilevich G Gennari L Salazar M Graziano C Saravia E Belinky J. Charlson Score as prognostic factor in patients with castration-resistant prostate cancer. J Clin Onco. (2017) 35:242–42. doi: 10.1200/JCO.2017.35.6_suppl.242

[9] Lieffers JR Baracos VE Winget M Fassbender K. A comparison of Charlson and Elixhauser comorbidity measures to predict colorectal cancer survival using administrative health data. Cancer. (2011) 117:1957–65. doi: 10.1002/cncr.25653 21509773

[10] Wagner D Marsoner K Tomberger A Haybaeck J Haas J Werkgartner G. Low skeletal muscle mass outperforms the Charlson Comorbidity Index in risk prediction in patients undergoing pancreatic resections. Eur J Surg Oncol. (2018) 44:658–63. doi: 10.1016/j.ejso.2018.01.095 29428474

[11] Charlson ME Pompei P Ales KL MacKenzie CR. A new method of classifying prognostic comorbidity in longitudinal studies: development and validation. J Chronic Dis. (1987) 40:373–83. doi: 10.1016/0021-9681(87)90171-8 3558716

[12] Eichhorn F Klotz LV Bischoff H Thomas M Lasitschka F Winter H. Neoadjuvant anti-programmed Death-1 immunotherapy by Pembrolizumab in resectable nodal positive stage II/IIIa non-small-cell lung cancer (NSCLC): the NEOMUN trial. BMC Cancer. (2019) 19:413. doi: 10.1186/s12885-019-5624-2 31046714 PMC6498462

[13] Shitara K Rha SY Wyrwicz LS Oshima T Karaseva N Osipov M. Neoadjuvant and adjuvant pembrolizumab plus chemotherapy in locally advanced gastric or gastro-oesophageal cancer (KEYNOTE-585): an interim analysis of the multicentre, double-blind, randomised phase 3 study. Lancet Oncol. (2024) 25:212–24. doi: 10.1016/s1470-2045(23)00541-7 38134948

[14] Yelena YJ Jaffer AA Markus M Lin S Marcelo G Carlos G. First-line nivolumab plus chemotherapy for advanced gastric, gastroesophageal junction, and esophageal adenocarcinoma: 3-year follow-up of the phase III checkMate 649 trial. J Clin Onco. (2024) null:JCO2301601. doi: 10.1200/jco.23.01601 PMC11185916 38382001

[15] Brahmer JR Tykodi SS Chow LQM Hwu W-J Topalian SL Hwu P. Safety and activity of anti-PD-L1 antibody in patients with advanced cancer. New Engl J Med. (2012) 366:2455–65. doi: 10.1056/NEJMoa1200694 PMC3563263 22658128

[16] Royal RE Levy C Turner K Mathur A Hughes M Kammula US. Phase 2 trial of single agent Ipilimumab (anti-CTLA-4) for locally advanced or metastatic pancreatic adenocarcinoma. J Immunotherapy (Hagerstown Md: 1997). (2010) 33:828–33. doi: 10.1097/CJI.0b013e3181eec14c PMC7322622 20842054

[17] Kate MB Michael B. Charlson comorbidity index guides treatment decisions for HER2 positive breast cancer in older women. J Am Coll Surgeons. (2021) 233 (5):e74–5. doi: 10.1016/j.jamcollsurg.2021.08.201

[18] Suzuki H Hanai N Nishikawa D Fukuda Y Koide Y Kodaira T. The Charlson comorbidity index is a prognostic factor in sinonasal tract squamous cell carcinoma. Japanese J Clin Oncol. (2016) 46:646–51. doi: 10.1093/jjco/hyw049%J 27162318

[19] Koseki Y Hikage M Fujiya K Kamiya S Tanizawa Y Bando E. Utility of a modified age-adjusted Charlson Comorbidity Index in predicting cause-specific survival among patients with gastric cancer. Eur J Surg Oncol. (2021) 47:2010–15. doi: 10.1016/j.ejso.2021.01.026 33558122

[20] Kang HW Kim SM Kim WT Yun SJ Lee SC Kim WJ. The age-adjusted Charlson comorbidity index as a predictor of overall survival of surgically treated non-metastatic clear cell renal cell carcinoma. J Cancer Res Clin Oncol. (2020) 146:187–96. doi: 10.1007/s00432-019-03042-7 PMC11804378 31606760

[21] Asano T Yamada S Fujii T Yabusaki N Nakayama G Sugimoto H. The Charlson age comorbidity index predicts prognosis in patients with resected pancreatic cancer. Int J Surg. (2017) 39:169–75. doi: 10.1016/j.ijsu.2017.01.115 28161529

[22] Onur İD Mutlu E Sertesen E Önder T Duran AO İnanç M. Evaluating the effectiveness of the Charlson Comorbidity Index in predicting immune checkpoint inhibitor-related adverse events. Immunotherapy. (2024) 16:295–303. doi: 10.2217/imt-2023-0270 38288692

[23] Andrew TF Heidi DK Beverly L Alexander MQ Chance HB Lara MK. Impact of comorbidity burden on immune-related adverse events and survival among older adults with cancer on immunotherapy. J Clin Oncol. (2023) 41 (16_suppl):12061. doi: 10.1200/jco.2023.41.16_suppl.12061

[24] Vincent de G Heleen B Gustaaf JL Lex MB. How to measure comorbidity: a critical review of available methods. J Clin Epidemiol. (2004) 57 (3):323. doi: 10.1016/j.jclinepi.2003.09.002 12725876

[25] Zhou S Zhang XH Zhang Y Gong G Yang X Wan WH. The age-adjusted charlson comorbidity index predicts prognosis in elderly cancer patients. Cancer Manag Res. (2022) 14:1683–91. doi: 10.2147/cmar.S361495 PMC9091471 35573259

[26] Henson SM Macaulay R Riddell NE Nunn CJ Akbar AN. Blockade of PD-1 or p38 MAP kinase signaling enhances senescent human CD8(+) T-cell proliferation by distinct pathways. Eur J Immunol. (2015) 45:1441–51. doi: 10.1002/eji.201445312 25707450

[27] Lages CS Lewkowich I Sproles A Wills-Karp M Chougnet C. Partial restoration of T-cell function in aged mice by *in vitro* blockade of the PD-1/ PD-L1 pathway. Aging Cell. (2010) 9:785–98. doi: 10.1111/j.1474-9726.2010.00611.x PMC2941565 20653631

[28] Patel SP Kurzrock R. PD-L1 expression as a predictive biomarker in cancer immunotherapy. Mol Cancer Ther. (2015) 14:847–56. doi: 10.1158/1535-7163.Mct-14-0983 25695955

[29] Mizrahi JD Surana R Valle JW Shroff RT. Pancreatic cancer. Lancet. (2020) 395:2008–20. doi: 10.1016/s0140-6736(20)30974-0 32593337

[30] Sung H Siegel RL Rosenberg PS Jemal A. Emerging cancer trends among young adults in the USA: analysis of a population-based cancer registry. Lancet Public Health. (2019) 4:e137–e47. doi: 10.1016/S2468-2667(18)30267-6 30733056

[31] Cai J Chen HD Lu M Zhang YH Lu B You L. Trend analysis on morbidity and mortality of pancreatic cancer in China, 2005-2015. Zhonghua Liu Xing Bing Xue Za Zhi. (2021) 42:794–800. doi: 10.3760/cma.j.cn112338-20201115-01328 34814469

