# Supplementary material for: Charlson syndrome index predicted survival in pancreatic cancer patients received immunotherapy
Source: Front Immunol. 2025 Feb 3;16:1487318. doi: 10.3389/fimmu.2025.1487318 (PMC11831428; doi:10.3389/fimmu.2025.1487318)
Supplement: Supplementary file 1 [file DataSheet1.docx]

**Appendix 1: Comorbidity Component and scoring**

| **Comorbidity Component** | **scoring** |
| --- | --- |
| Myocardial Infarction | 1 |
| Congestive Heart Failure | 1 |
| Peripheral Vascular Disease | 1 |
| Cerebrovascular Disease | 1 |
| Dementia | 1 |
| COPD | 1 |
| Connective Tissue Disease | 1 |
| Peptic Ulcer Disease | 1 |
| Diabetes Mellitus | 1 point uncomplicated, 2 points if end-organ damage |
| Moderate to Severe Chronic Kidney Disease | 2 |
| Hemiplegia | 2 |
| Leukemia | 2 |
| Malignant Lymphoma | 2 |
| Solid Tumor | 2 points, 6 points if metastatic |
| Liver Disease | 1 point mild score，3 points if moderate and severe score |
| AIDS | 6 |

The specific judgements principles are as follows:

**1.Myocardial Infarction:** 1point for a history of myocardial infarction, confirmed by elevated myocardial enzyme levels;

**2.Congestive Heart Failure:** 1 point for reduced ejection fraction, decreased exercise tolerance, and elevated BNP levels, confirmed by echocardiography;

**3.Peripheral Vascular Disease:** 1 point for venous thrombosis or vascular stenosis, confirmed by peripheral vascular ultrasound;

**4.Cerebrovascular Disease**: 1 point for history of hemorrhagic or ischemic encephalopathy;

**5.Dementia**: no patients had dementia, scored 0 point;

**6.COPD (chronic obstructive pulmonary disease):** 1 point for Chronic cough and expectoration for more than 3 months annually;

**7.Connective Tissue Disease**: 1 point for Rheumatoid arthritis, Sjogren's syndrome or systemic sclerosis, the rest of the connective tissue diseases were not seen in this study group. Confirmed by medical history and related antibodies;

**8.Peptic Ulcer Disease**: 1 point with stomach pain and acid reflux confirmed by gastroscopy;

**9.Diabetes Mellitus**:1 point without complications; 2 points with complications, including microvascular diseases such as diabetic foot and macrovascular diseases involving vital organs);

**10.Moderate to Severe Chronic Kidney Disease**:1 point for moderate-to-severe impairment based on creatinine levels;

**11.Hemiplegia:** 1 point for unilateral limb hemiplegia and lower limb movement disorders;

**12.Leukemia:**1 point for a history of leukemia;

**13. Malignant Lymphoma:**1 point for a history of malignant lymphoma;

**14.Solid Tumor**: 2 points; 6 points if metastatic;

**15.****Liver Disease:** 0 point for Child-Pugh A, 1 point for Child-Pugh B and 3 point for Child-Pugh C. The classification is based on the Child-Pugh score, which evaluates hepatic encephalopathy, ascites, total bilirubin, albumin level and prolonged prothrombin time;

**16.AIDS**: no patients had AIDS, scored 0 point.
